# SGM-DETR: Semantic-Guided and Feature-Refined Transformer for Pine Wilt Disease Detection in Satellite Imagery

**DOI:** 10.3390/plants15131959

**Published:** 2026-06-25

**Authors:** Xixin Chen, Zidi Wu, Zhuangci Wu, Xiaobo Tan, Yongfei Xue, Yuanhan Luo, Peng Wang, Wenjing Huang, Jianhua He, Jie Zhang, Jizheng Yi

**Affiliations:** College of Computer and Mathematics, Central South University of Forestry and Technology, Changsha 410004, China; 20232140@csuft.edu.cn (X.C.); 20241200780@csuft.edu.cn (Z.W.); 20233669@csuft.edu.cn (Z.W.); 20241200783@csuft.edu.cn (X.T.); t20252936@csuft.edu.cn (P.W.); t20142191@csuft.edu.cn (W.H.); 20231792@csuft.edu.cn (J.H.); 20241100477@csuft.edu.cn (J.Z.); t20152279@csuft.edu.cn (J.Y.)

**Keywords:** pine wilt disease, satellite remote sensing, semantic–visual fusion, vegetation index, RT-DETR, object detection, Stackelberg game optimization

## Abstract

Pine wilt disease (PWD) can spread rapidly after the disease occurs and often causes large-scale death of the pine. Therefore, the timely identification of infected trees is critical for forest conservation and effective disease management. However, early infected trees are difficult to distinguish in satellite remote sensing images. Their visual differences from healthy trees and complex background features are often subtle, and existing image-processing methods do not fully exploit heterogeneous information. To address this problem, we constructed the Naro dataset for satellite-based PWD detection and proposed SGM-RTDETR based on Real-Time Detection Transformer (RT-DETR). The proposed model consists of a Semantic–Visual Fusion Module (SVFM) and a Disease Feature Refinement Module (DFRM). In SVFM, ExG, VARI, and GLI are concatenated with RGB imagery to form a six-channel visual input, which enhances the spectral differences between diseased and non-diseased targets. In addition, textual prior knowledge is introduced into the decoder input through a Stackelberg game-based visual–text fusion strategy. This strategy helps the encoded memory features maintain clearer disease-related semantics in complex backgrounds. DFRM then performs channel recalibration, feature refinement, and residual enhancement on the fused memory features to better extract fine-grained disease cues in remote sensing scenes. Experiments on the Naro dataset show that SGM-RTDETR achieves 80.75% mAP@0.5 and 35.43% mAP@0.5:0.95, which is 2.74 percentage points higher than RT-DETR-L on mAP@0.5:0.95. Overall, the results indicate that the dual-module structure improves the precision and robustness of PWD detection in satellite remote sensing images.

## 1. Introduction

A pine forest is a coniferous forest in a warm or cool–temperate and subtropical region of China. The ecology of these is wide-ranging. They strengthen the regulation of water, carbon and nitrogen fixation in the soil, improve soil conditions, reduce erosion, and enhance biodiversity [1]. Pine forests are also sources of timber, resin and other raw materials for the chemical industry, as well as the ecological services listed above [2]. Therefore, these two are closely related to the stability of the environment and the economic development of mountainous forest areas; among the biological hazards affecting pine forests, pine wilt disease (PWD) is particularly serious [3]. PWD is a serious forest disease caused by the pine wood nematode Bursaphelenchus xylophilus, and an insect vector for its transmission is the pine sawyer beetle Monochamus alternatus [4]. A tree that has been infected with disease will be unable to transport water and nutrients to the rest of the tree efficiently. Generally, as a result of the above, the crown will yellow; it will become gradually weakened and eventually die [5]. As the disease spreads to cover more areas and affects more trees, the damage may also reach all the trees in the forest and cause severe harm to the forest. Traditional monitoring is still mainly done through field observation and experience. These approaches are still feasible for forestry work in practice, but they require considerable labor and time. They are also not suitable for applying in early infection detection across a wide area of the forest uniformly and promptly. Therefore, in time, the damaged trees affected by PWD need to be located, diseased people should be found, the range of the outbreak determined, the transmission paths cut off, infected trees removed, and support for prevention and control provided. This demand is relatively high at the beginning of the infection. At this time, high-speed, high-reliability recognition technology will improve monitoring efficiency, reduce missed and false detections, and prevent further ecological and economic damage. Therefore, this paper will focus on the automatic detection of PWD from satellite remote sensing images for large-scale forest health monitoring [6].

Forestry staff have been conducting field-based visual inspections for a long time to survey damaged forests. Although this way is simple to understand and directly usable, it is not suitable for a large-area application. The effect of this will be limited by the lack of labor, the inaccessibility of the land, and other physical difficulties. There are many scattered full-coverage surveys in the large mountain forest area, and we cannot react promptly to disease outbreaks. Remotely sensed data may be available for this purpose [7]. A UAV can take a high-definition, near-ground photograph from above [8]. However, due to its short flight time and small coverage area, it is not practical for the large-scale, continuous monitoring of many places. Satellite remote sensing is suitable for observing the widespread forest area in a region because it has a large spatial extent, a relatively high revisit frequency, and stable multi-band observation capabilities [9]. With the development of high-resolution satellite systems such as Beijing-3 and WorldView, many more observations of forest areas over a large area have been realized. RGB satellite images can be used to get visible-band vegetation indices such as VARI, GLI and ExG [10]. Although these indicators do not contain near-infrared data, they can still show the health and stress of the canopy to some extent [11]. Therefore, they offer some reference points for identifying trees damaged by pine wood nematode. At the same time, deep learning has also been used to automatically recognize PWD symptoms [12]. Deep neural networks can learn discriminative patterns directly from image data, unlike methods based on hand-crafted features, and are generally more suitable for complex forest backgrounds [13]. Based on the above, this paper will use satellite remote sensing, visible-spectrum vegetation indices and deep learning-based object detection to identify PWD-infected trees automatically [14].

Most of the deep learning-based object detection methods fall into the two-stage and one-stage frameworks. A two-stage detector generates candidate regions first, and then classifies and regresses bounding boxes [15]. We design R-CNN-style models, which are good at localizing, as shown above [16]. However, the multi-step procedure is also computationally expensive and therefore less suitable for time-sensitive applications of remote sensing data. A single-stage detector directly predicts object classes and bounding boxes in one forward pass [17]. They have a simple inference pipeline and are thus relatively high performance; they are widely used for real-time and large-scale detection tasks [18]. The YOLO family is a typical case of this development. YOLOv3 used an optimized Darknet backbone and was then applied to PWD detection. YOLOv4 and YOLOv5 enhanced multi-scale feature extraction through BoF/BoS strategies and feature pyramids. YOLOv7 focused more on lightweight inference, and YOLOv8 is an anchor-free design using a C2f-based head to enhance both accuracy and speed [19]. YOLOv11 has been enhanced with modules such as C3k2 and C2PSA in recent times to improve detection performance further. Although there have been many improvements, CNN-based detectors are still relatively limited in modeling long-range spatial correlations of satellite forest imagery because of the local receptive field nature of convolution. Therefore, transformer-based detectors have recently been introduced [20]. DETR is an encoder–decoder Transformer that has been introduced to address the problem of manual anchor design and non-maximum suppression in object detection [21]. RT-DETR has increased the above framework by adding a hybrid encoder and an efficient query selection mechanism to address both convergence issues and the computational cost of the previous DETR-like models [22]. Among the above architectures, RT-DETR-L combines the HGNetv2 backbone and a Transformer encoder, and is suitable for satellite image analysis that requires both local detail perception and broader contextual understanding. Therefore, RT-DETR-L is chosen as the baseline model for further optimization in this study.

**Figure 1:**
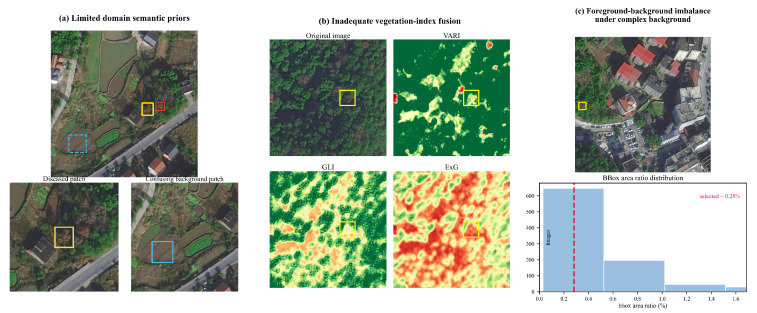
Typical challenges of pine wilt disease detection in satellite remote sensing scenarios.

Although some progress has been made in the development of PWD detection models recently, accurately detecting PWD-infected pine trees in satellite remote-sensing images remains challenging. First, many of the available methods are mainly based on pixel-level visual features and do not extensively utilize disease-related prior knowledge. Such knowledge may include alterations in canopy shape during the development of the disease, indicators of plant health, and spatial distributions of disease spread. Without such information, early-infected trees may be missed; especially when the symptoms are mild and the visual difference in a healthy and diseased tree’s canopy is small, they are even harder to spot. Second, although the various spectral information in satellite images can be employed, vegetation indices are often used only as supplementary inputs. They are not always closely related to the spatial texture features, and thus the combined discriminatory power of spectral and visual cues is relatively low. Third, remote sensing data generally contain a larger number of healthy trees than diseased ones. This imbalance may cause the training process to favor the dominant background or healthy samples, reduce the model’s sensitivity to minority diseased targets, and thus lower localization stability [23]. The above problems are shown in Figure 1. As shown in Figure 1a, without disease-specific semantic guidance, infected trees and visually similar healthy backgrounds may have the same colors and textures, thus increasing the risk of confusion. Figure 1b shows that diseased trees may not be sufficiently distinguishable in the original RGB image, whereas vegetation indices such as VARI, GLI, and ExG provide different responses to canopy damage, indicating that these vegetation-index cues should be effectively fused with RGB spatial features rather than used only as isolated auxiliary information. Figure 1c also shows that diseased tree crowns occupy only a small proportion of complex remote-sensing scenes as reflected by the bounding-box area-ratio distribution, resulting in a very uneven foreground–background distribution and making minority-target detection more challenging. Together, these examples show that reliable satellite-based PWD detection needs a model with enhanced vegetation-related signal strengthening, integration of disease-relevant semantic information, and retention of fine local target details [24]. Thus, a research gap has been identified: although there are existing satellite-based PWD detectors, they do not yet have an effective end-to-end mechanism for the joint exploitation of vegetation-index information, domain semantic knowledge, and fine-grained local features. To address the above problem, SGM-RTDETR based on RT-DETR-L is proposed in this paper. SVFM and DFRM are introduced in the proposed framework to enhance feature representation and discrimination ability for diseased target plants in complex forest environments through multi-dimensional clustering design. The objective of this study is to improve the accuracy and robustness of PWD detection in satellite images, expand its application scope, and reduce deployment costs. We hypothesize that integrating vegetation-index enhancement, disease-related semantic guidance, and refined local-detail modeling into RT-DETR-L can improve the discrimination and localization of PWD-infected trees in complex forest scenes.

Given these limitations, we place emphasis on three specific problems in satellite-based PWD detection. First, disease-related prior knowledge needs to be better used. Second, vegetation-index cues should be combined more closely with visual features. Third, weak symptoms must be distinguished more reliably in complex forest scenes. On this basis, RT-DETR-L was adopted as the baseline detector because it provides a favorable balance between accuracy and efficiency. We further developed SGM-RTDETR by combining semantic enhancement with feature refinement. The main contributions are listed below.

(a) A high-resolution satellite remote sensing annotated dataset, named Naro, is constructed specifically for PWD detection. This dataset contains 952 professionally annotated satellite images covering infected pine tree targets against complex forest backgrounds, providing reliable data support and an evaluation benchmark for PWD detection research in satellite remote sensing scenarios.

(b) A Semantic–Visual Fusion Module (SVFM) is proposed. Through vegetation index-guided visual enhancement and Stackelberg game-based visual–textual collaborative fusion, spectral information and domain-specific semantic priors are introduced at the input stage and the decoder input stage, respectively, enabling hierarchical complementary modeling of multi-source information and thereby enhancing the discriminative capability for disease targets under complex backgrounds.

(c) A Disease Feature Refinement Module (DFRM) is proposed. This module operates on the encoder memory feature pathway and further optimizes the SVFM-augmented features through three successive stages: channel recalibration, local detail refinement, and residual enhancement. This design emphasizes channel recalibration in highlighting the more important information about the disease features, channel local refinement in enhancing the depiction of minute lesions and minimal structural differences, and residual improvement in maintaining stability in the transmission of features and retaining valuable original material. Consequently, the model has become more efficient in recognizing and pinpointing more subtle disease patterns in complicated remote sensing scenes.

## 2. Materials and Methods

### 2.1. Dataset Collection

Through the technical assistance of the Forestry Survey and Monitoring Center of Hunan Province, China, we established a satellite remote sensing dataset for pine wilt disease (PWD) detection, named Naro. The remote sensing images were acquired from major PWD surveillance zones in south-central Hunan Province, which is located in a subtropical monsoon climate region. The study area is mainly covered by Masson pine (Pinus massoniana) and slash pine (Pinus elliottii) forests, intermixed with broadleaf stands, and pine forests account for more than half of the total forest cover. Because PWD is widely distributed in this region, it provides representative conditions for developing and evaluating remote sensing-based PWD detection methods. In addition, the forest structure is complex, with canopy, mid-story, and understory layers in the vertical direction and large spatial variation in tree density in the horizontal direction, resulting in diverse and challenging background conditions.

**Figure 2:**
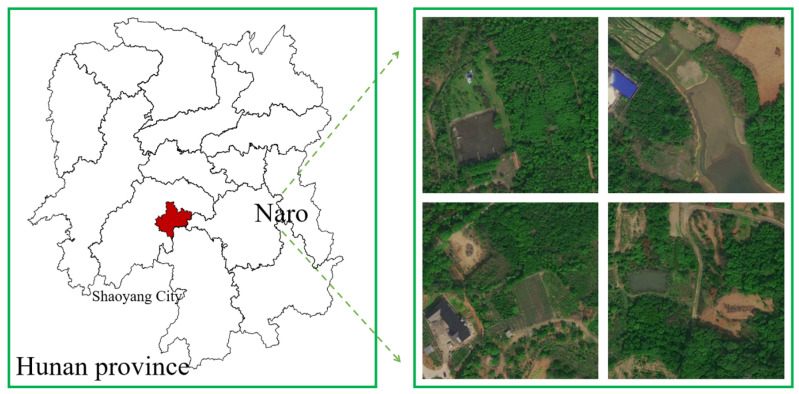
Dataset collection process.

In September and October 2024, images were acquired. At this time of year, pine trees that have been affected by PWD generally show obvious decline symptoms, and their crowns are changing color from yellow-brown to reddish-brown. Healthy broad-leaved trees in the vicinity are typically quite large and regularly shaped. Therefore, this phenological difference can be used to prepare samples for PWD recognition. The remote sensing images were acquired by the Beijing-3 (BJ-3) satellite, which provides 0.5 m panchromatic resolution, 2 m multispectral resolution, a 23 km imaging swath, and visible RGB bands. To identify diseased pine crowns with a sub-meter spatial database, R is used to calculate visible-band vegetation indices such as VARI, GLI and ExG. The entire process of data collection is as follows in Figure 2.

As the scenes of each individual in BJ-3 are relatively large, a sliding-window cropping method was used to obtain 640 × 640 pixel patches from the original images. To improve the quality and reliability of the samples and annotations, forestry experts then manually inspected the generated patches. Images with cloud cover, haze, strong shadows or severe blur were excluded from the next step. After the above quality control, 952 valid image patches were obtained for construction of the Naro dataset. The above samples are representative of several background categories, including bare ground, broad-leaved forests, dead trees and PWD-infected pines. Then, the entire set of images was randomly divided into training, validation and test sets at the image level instead of dividing individual bounding boxes. Approximately speaking, the split ratios were 7:2:1; therefore, there were about 666 training images, 190 validation images, and 96 test images. Annotations were carried out in each subset after splitting, and thus bounding-box instances from the same image did not appear in different data subsets.

Forestry personnel with practical experience in recognizing PWD symptoms manually marked the infected tree crowns in LabelImg 1.8.6. The annotation files were exported in TXT format, with bounding-box coordinates normalized to the image size. In total, the dataset contained 2328 annotated PWD tree instances, including 1616 instances in the training set, 504 instances in the validation set, and 208 instances in the test set. The annotated PWD samples include infected pine trees with different symptom severities, including early, middle, and late stages. However, during annotation, these samples were labeled as one unified diseased-tree class rather than being divided into stage-specific categories.

### 2.2. SGM-RTDETR

**Figure 3:**
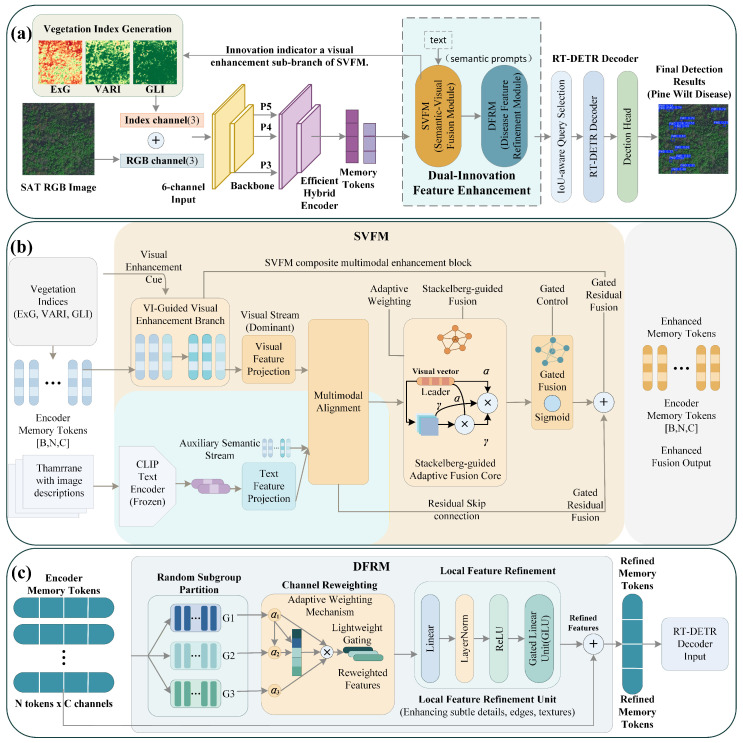
Overall architecture of SGM-RTDETR. (**a**) Complete network structure of the backbone–encoder–decoder pipeline; (**b**) internal structure of the SVFM module; (**c**) schematic of the DFRM optimization process.

Built on RT-DETR-L, SGM-RTDETR introduces two modules, SVFM and DFRM, to strengthen PWD target detection while keeping the original end-to-end detection pipeline compact and efficient as illustrated in Figure 3. The overall pipeline is as follows: First, during the input preprocessing stage, three visible-band vegetation indices—ExG, VARI, and GLI—are computed from the original RGB image and concatenated with it to form a 6-channel input. Subsequently, the 6-channel image is first processed by HGNetv2 to capture visual features at multiple scales, after which the HybridEncoder performs global context modeling and cross-scale fusion to generate the encoded memory features. Next, before the memory features are fed into the RT-DETR decoder, SVFM first adaptively enhances the encoder memory features using CLIP textual semantics through a Stackelberg game-based mechanism. The resulting SVFM-enhanced encoder memory features are then used as the direct input of DFRM, which further applies channel recalibration, local refinement, and residual enhancement. Finally, the enhanced memory features are fed into the RT-DETR decoder to produce the detection results.

#### 2.2.1. Semantic–Visual Fusion Module (SVFM)

In the satellite remote sensing detection task for PWD, relying solely on RGB visual features often fails to reliably distinguish between healthy trees, infected trees, and similar interfering targets in complex backgrounds. On the one hand, the canopy color of trees at the early stage of PWD infection is highly similar to that of healthy trees, making reliable detection difficult using only visible-band information [25]. On the other hand, the spectral responses of non-target land cover elements in complex forest backgrounds, such as bare soil, dead branches, and rocks, overlap with those of infected tree canopies, readily triggering false detections [26]. To address this, we propose the Semantic–Visual Fusion Module (SVFM), which synergistically enhances feature representation from two complementary perspectives: visual enhancement and semantic guidance. Specifically, the SVFM comprises a vegetation index-guided visual enhancement sub-branch and a Stackelberg game-based visual–textual fusion module. First, during the input preprocessing stage, three visible-band vegetation indices—ExG, VARI, and GLI—are computed from the RGB image and concatenated with the original RGB image to form a 6-channel visual input, enabling the backbone network to explicitly perceive vegetation health status differences at the early stage of feature extraction [27]. Then, the pre-computed and cached CLIP text embeddings are applied on the decoder input to carry out adaptive improvement of the memory feature encoded in the training stage [28]. We will use a gated residual framework, which is based on a Stackelberg game, to combine both visual and text data more uniformly [29]. This is done in such a way that the visual indicators are dominant, whereas the text indicators are auxiliary guiding indicators and the learnable weights can control their relative impact to ensure that slight signs of illness can be more effectively differentiated against complex backgrounds.

The internal structure of the SVFM is depicted in Figure 3b, and its particular processing scheme is as shown below. Initially, each input image is encoded into a 512-dimensional text embedding vector, with the corresponding domain-specific textual description being encoded by a pre-trained CLIP model, and the resulting vector is projected into the space of the visual features via a linear projection layer. In this case, T represents the original CLIP text embedding, $W_{p}$ represents the projection weight matrix, LN represents layer normalization, and GELU represents the activation function:

(1) $$F_{t}=\mathrm{GELU}\left(\mathrm{LN}(W_{p}\times T)\right)\in R^{B\times C}$$

Subsequently, the projected text features are broadcast-expanded along the sequence dimension to match the shape of the encoder output visual features $F_{v}\in R^{B\times N\times C}$, where *B* denotes the batch size, *N* denotes the number of encoded spatial tokens or memory positions generated by the encoder, and *C* denotes the feature channel dimension. The expanded text features are then concatenated with the visual features along the channel dimension before being fed into a fusion gating network to compute element-wise fusion weights. Here, $F_{t}^{↑}$ is obtained by broadcast-expanding the text features. The notation [·;·] means that features are concatenated along the channel dimension, and $W_{g}$ refers to the weight used in the gating network:

(2) $$F_{t}^{↑}=\mathrm{Expand}\left(F_{t},N\right)\in R^{B\times N\times C}$$

(3) $$g=\sigma \left(\mathrm{LN}\left(W_{g}\;\left[F_{v};F_{t}^{↑}\right]\right)\right)\in R^{B\times N\times C}$$

The interaction between the visual and textual features is further described using a Stackelberg game. Here, the visual features are regarded as the Leader, as they retain the main disease-related cues. The textual features are used as the Follower and provide complementary semantic information. The game parameters $\hat{\alpha }$ and $\hat{\beta }$ are learnable scalars, with initial values set to 0.7 and 0.3, respectively, and are mapped to strategy coefficients α and β within the (0,1) interval through the Sigmoid function. Here, α represents the influence weight of the visual leader, and β represents the response weight of the textual follower. Their product γ=α·β is used as the game equilibrium coefficient, which controls the overall strength of textual semantic guidance injected into the visual memory features in the residual fusion process:

(4) $$\alpha =\sigma \left(\hat{\alpha }\right),\;\beta =\sigma \left(\hat{\beta }\right),\gamma =\alpha \cdot \beta$$

Finally, the fused output is obtained through a residual connection. Here, λ is a learnable residual scaling factor (initialized to 0.1), and ⊙ denotes element-wise multiplication. The vegetation indices have already been incorporated as additional channels into the backbone network input during the data processing stage, and are implicitly fused with the visual features $F_{v}$ extracted by the encoder:

(5) $$F_{\mathrm{out}}=F_{v}+\lambda \;\gamma \;\left(g⊙F_{t}^{↑}\right)$$

Through the dual mechanism of vegetation index-guided visual enhancement and Stackelberg game-driven semantic fusion, the SVFM module enables the model to explicitly perceive vegetation health status differences at the early stage of feature extraction, and adaptively augment the encoded features with textual semantic priors during the decoding phase, thereby accurately identifying dead and diseased regions associated with PWD in satellite remote sensing imagery. Even under scenarios with complex mountainous background interference and significant target scale variations, the model can efficiently capture subtle spectral and textural differences between healthy and infected trees through the gated residual fusion and game equilibrium weighting mechanisms. This approach effectively addresses the problems of insufficient utilization of domain-specific semantic priors and inadequate vegetation index information fusion in satellite remote sensing scenarios, and provides robust semantic discrimination and fusion capabilities for multi-source feature representation of disease targets.

#### 2.2.2. Disease Feature Refinement Module (DFRM)

To better handle subtle disease cues under complex background conditions, DFRM is placed after SVFM and takes the SVFM-enhanced encoder memory features as its direct input. The design draws on the ideas of random subgroup partitioning, attraction aggregation, and purification, but instead of replacing training optimizers such as AdamW, it is implemented as a lightweight neural module for refining encoder memory features. In this module, random subgroup partitioning is reflected by the channel recalibration branch, which assigns different importance weights to different channel dimensions; attraction aggregation is implemented through the nonlinear refinement branch applied to the attention-weighted features; and purification is achieved by the zero-initialized residual correction path, which gradually learns stable feature updates while avoiding destructive interference. Given the SVFM-enhanced encoder memory feature *X*, DFRM keeps an identity mapping during the warmup stage at the beginning of training so that the early convergence of the backbone and encoder is not disturbed. Once warmup is finished, the module first evaluates the relative importance of each channel through a channel recalibration branch, then obtains the attention-weighted feature $X_{\mathrm{att}}$, applies a nonlinear transformation to $X_{\mathrm{att}}$ in a feature refinement branch, and finally adds the refined response *R* back to the original input feature *X* through a residual path, with the enhancement strength controlled by a learnable scaling factor. Thus, the module adds sensitivity to disease-related textures, edges, and local structural variations without disturbing the valuable information already present in the SVFM-enhanced encoder memory features.

Figure 3c indicates that DFRM uses the SVFM-enhanced encoder memory features as its direct input, represented as $X\in R^{B\times N\times C}$. During the initial three epochs of training, the module keeps an identity mapping and directly passes these features to the decoder to maintain stable early optimization. After this warmup stage, DFRM begins to refine the features through four successive steps.

First, a channel attention mechanism is applied to the original input feature *X* to evaluate the relative importance of the *C* channels and to generate channel-wise attention weights *A*. In effect, this process corresponds to the random subgroup partitioning idea, because channels are implicitly grouped according to different importance levels so that more disease-relevant feature dimensions receive greater emphasis. Here, $W_{1}\in R^{C/4\times C}$ and $W_{2}\in R^{C\times C/4}$.

(6) $$A=\sigma \left(W_{2}\;\mathrm{ReLU}\left(W_{1}X\right)\right)\in R^{B\times N\times C}$$

(7) $$X_{\mathrm{att}}=X⊙A$$

Next, the attention-weighted feature $X_{\mathrm{att}}$ is fed into a two-layer fully connected refinement network, where more discriminative feature representations are extracted through layer normalization and ReLU nonlinear transformation. This step corresponds to attraction aggregation, because the recalibrated features are further transformed toward a more disease-discriminative representation direction. Here, $W_{3}\in R^{C\times C}$ and $W_{4}\in R^{C\times C}$; the refinement branch does not adopt a bottleneck structure in order to preserve the full channel expressiveness.

(8) $$R=W_{4}\;\mathrm{ReLU}\left(\mathrm{LN}(W_{3}X_{\mathrm{att}})\right)\in R^{B\times N\times C}$$

Subsequently, the last layer of the refinement network adopts a zero initialization strategy, with both weights and biases initialized to 0. This causes the refinement branch to output near-zero correction vectors at the early stage of training, thereby avoiding destructive interference with the SVFM-enhanced encoder memory features. This step corresponds to the purification idea, because noisy or unstable correction signals are suppressed at the beginning of training and meaningful feature correction signals are gradually learned as training progresses.

Finally, the feature correction intensity is controlled through a learnable scaling factor *s*, where its raw parameter $\hat{s}$ is initialized with a small value and mapped through the Sigmoid function before participating in the residual output. After obtaining the refined response *R*, we scale it and add it to the input feature *X* to generate $X_{\mathrm{out}}$. In this way, the output still retains information from the original SVFM-enhanced encoder memory features.

(9) $$s=\sigma (\hat{s})$$

(10) $$X_{\mathrm{out}}=X+s\;R$$

DFRM refines the SVFM-enhanced encoder memory features through three successive steps: channel subgroup partitioning, feature attraction aggregation, and zero-initialized purification. With this design, the model can give more attention to disease-related channel responses while avoiding unnecessary disturbance to the representations that have already been learned. This is useful for distinguishing weak texture and edge variations between dead pine trees and nearby healthy vegetation in satellite images. In the presence of adverse circumstances, such as canopy occlusion in mountainous areas and shadow interference, residual scaling enhancement and warmup protection can be employed to extract subtle disease signals more reliably by the model. Therefore, DFRM can enhance the representation of fine-grained target characteristics in a dense forest setting and furnish a more robust feature refinement tool for high-accuracy PWD detection in satellite remote-sensing images.

## 3. Results

### 3.1. Experimental Environment and Training Details

To keep the comparisons consistent, all experiments were conducted under the same computing environment and software configuration. The detailed hardware and software specifications are listed in Table 1. The core training hyperparameters for the current dual-innovation experiment were set according to the final training script train_combined_vi_svfm_rslpo.py as follows: epochs = 100, batch = 4, imgsz = 640, optimizer = AdamW, lr0 = $1\times 10^{-4}$, lrf = 0.01, momentum = 0.937, weight_decay = $5\times 10^{-4}$, warmup_epochs = 3, patience = 50, workers = 0, and seed = 42. Automatic mixed precision (AMP) was enabled during training. In this study, workers = 0 means that data were loaded in the main process. This setting was used to maintain a stable and reproducible training environment under the current experimental platform and dataset scale, rather than to indicate an optimal data-loading configuration for larger-scale training scenarios.

For the newly introduced SVFM and DFRM parameters, the training script appends them as an independent optimizer parameter group with a reduced learning rate of 0.1×lr0, namely $1\times 10^{-5}$. This grouped learning-rate strategy was used to reduce the disturbance of the newly introduced modules to the pre-trained feature distribution during optimization. During training, several data augmentation strategies were used, including HSV color perturbation, random translation, random scaling with scale =0.5, random horizontal flipping with p=0.5, Mosaic augmentation, RandAugment, and random erasing with p=0.4. Specifically, Mosaic augmentation was applied with a probability of 1.0 during the first 90 epochs and was disabled during the last 10 epochs to facilitate model convergence. MixUp, CutMix, vertical flipping, shearing, rotation, and perspective transformation were not used.

The CLIP text embeddings used in SVFM were generated using the ViT-B/32 model and were pre-computed and cached for the domain-specific textual descriptions of each training image. Therefore, this text-embedding process introduced no additional text-encoding overhead during inference.

For quantitative and statistical analysis, no additional inferential statistical tests were conducted. Instead, all models were compared under the same dataset split, training configuration, and evaluation protocol using standard object detection metrics, including Precision, Recall, mAP@0.5, and mAP@0.5:0.95. Model complexity and deployment efficiency were further evaluated using Params, GFLOPs, and inference speed.

### 3.2. Evaluation Metrics

We assess detection performance using four metrics: mAP@0.5, precision, recall, and mAP@0.5:0.95. Their calculation formulas are presented below.

MAP is obtained by averaging AP across all categories. AP represents the area under the precision–recall curve and describes how precision and recall change as the confidence threshold varies. Because of this, mAP is commonly used to reflect the overall detection quality of a model. In our experiments, mAP@0.5 is computed at an IoU threshold of 0.5, whereas mAP@0.5:0.95 is calculated by averaging the results over IoU thresholds from 0.5 to 0.95 with an interval of 0.05:

(11) $$mAP=\frac{1}{N}\sum_{i=1}^{N}AP_{i}$$

Precision measures how accurately the model identifies positive targets. TP (True Positive) in the present research means infected pine trees that have been properly identified while FP (False Positive) refers to healthy pine trees that have been wrongly categorized as infected targets:

(12) $$\mathrm{Precision}=\frac{TP}{TP+FP}$$

Recall indicates the number of infected pine trees that the model can identify correctly. In this research, FN (False Negative) stands for infected pine trees that were wrongly classified as healthy targets:

(13) $$\mathrm{Recall}=\frac{TP}{TP+FN}$$

IoU (Intersection over Union) denotes the ratio between the overlapping area and the union area of a predicted bounding box $B_{\mathrm{pred}}$ and its matched ground-truth bounding box $B_{\mathrm{gt}}$. It is applied when determining whether a detection result can be considered a true positive:

(14) $$\mathrm{IoU}=\frac{\left|A\cap B\right|}{\left|A\cup B\right|}=\frac{\mathrm{Area}\;\mathrm{of}\;\mathrm{Overlap}}{\mathrm{Area}\;\mathrm{of}\;\mathrm{Union}}$$

In the equation above, $B_{\mathrm{pred}}$ denotes the predicted bounding box, and $B_{\mathrm{gt}}$ denotes the matched ground-truth bounding box. In every comparative experiment conducted in this paper, mAP@0.5 is the main evaluation measure and mAP@0.5:0.95 is a secondary measure so that the findings are consistent with the research carried out by the mainstream in this discipline.

### 3.3. Model Performance Analysis

In order to examine generalization, we have also considered a five-fold validation scheme based on samples. The data was divided into five subsets of equal size, and in every round, one of them served as a validation set and the remaining four were used to train. It was done until the five subsets were used once as a validation set. On every fold, the best model of that run was chosen and the overall accuracy was given as an average of the five folds.

**Figure 4:**
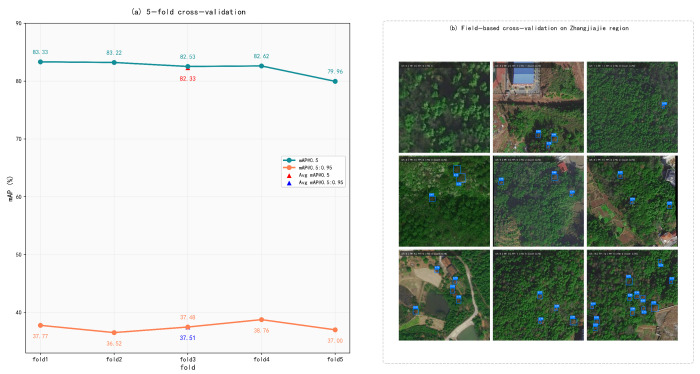
Cross-validation and representative detection results. (**a**) Five-fold cross-validation on the PWD satellite remote sensing dataset, where the points show fold-wise mAP@0.5 and mAP@0.5:0.95 values and the dashed lines indicate their mean values; (**b**) representative qualitative detection results in Zhangjiajie City, where blue boxes denote SGM-RTDETR detections under different site types.

Figure 4b demonstrates that SGM-RTDETR has similar performance at various data partition settings. The mAP@0.5 values on the five folds are 83.33% (fold 1), 83.22% (fold 2), 82.53% (fold 3), 82.62% (fold 4) and 79.96% (fold 5), which results in an average of 82.33% and a standard deviation of 1.23%. The respective mAP@0.5:0.95 values are 37.77 (fold 1), 36.52 (fold 2), 37.34 (fold 3), 38.76 (fold 4) and 37.00 (fold 5) with the average of 37.48 and the standard deviation of 0.76. Even though the fifth fold is slightly lower than the rest, the variation between the folds is still minimal. It implies that the model has great generalization strength and consistent performance in the case of random sampling.

In order to further test the spatial transferability of the model, we performed representative detection validation on satellite remote sensing images of various site types in Zhangjiajie City. The images were taken in the Yongding District and Cili County of Zhangjiajie City, Hunan Province (28.90°N–29.40°N, 111.65°E–111.80°E). This area is characterized by a variety of landscapes, such as dense coniferous forests, sparse mixed forests, farmland, and areas with a mixture of buildings and plants. Nine representative samples of various scene types were chosen for qualitative visual validation, as illustrated in Figure 4b, where the blue boxes indicate the detection results generated by SGM-RTDETR. It should be noted that Figure 4b is used to provide representative visual examples rather than an independent quantitative comparison; the quantitative performance of the model is evaluated using the cross-validation results in Figure 4a and the standard detection metrics reported in the comparative experiments. It can be seen that SGM-RTDETR is able to locate small numbers of targets in sparsely wooded areas, identify diseased pine tree targets in building–vegetation mixed-scene backgrounds, and maintain stable detection behavior in densely wooded areas with larger target populations and highly similar background textures. These findings indicate that the model is likely to have good prospects of being applied in a wide range of pine wilt disease detection settings.

**Figure 5:**
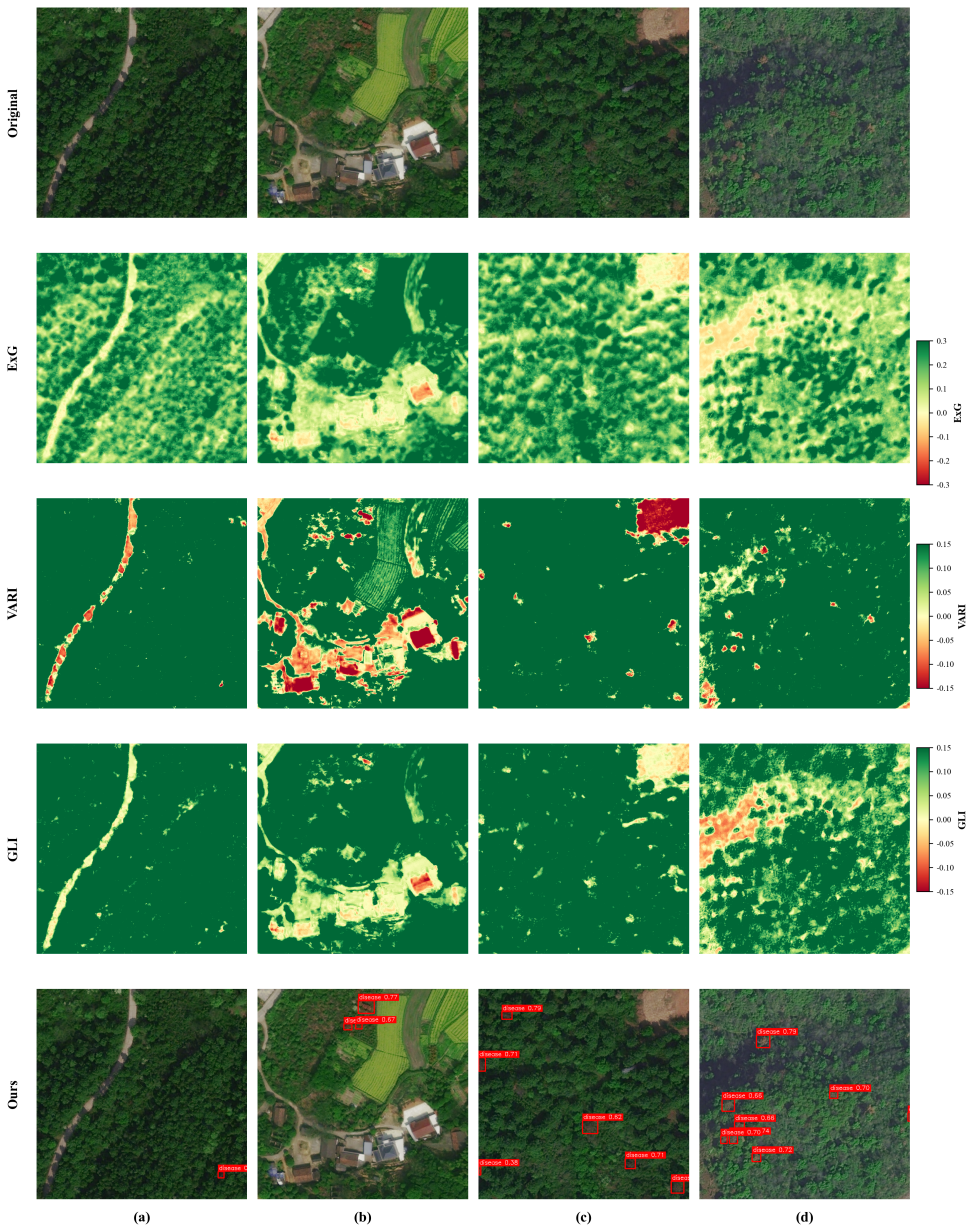
Vegetation indices and detection results. Columns (**a**–**d**) represent four representative remote sensing samples. Rows from top to bottom show the original RGB image, ExG map, VARI map, GLI map, and SGM-RTDETR detection result, respectively.

To make the detection results easier to interpret, Figure 5 displays four representative groups of results arranged by columns. From top to bottom, each row shows the original remote sensing RGB image, the ExG map, the VARI map, the GLI map, and the detection results produced by SGM-RTDETR. Unlike the conventional Normalized Difference Vegetation Index (NDVI), which depends on near-infrared information, this study uses vegetation indices computed only from visible RGB bands, namely the Excess Green Index (ExG), the Visible Atmospherically Resistant Index (VARI), and the Green Leaf Index (GLI). Their calculation formulas are given below:

(15) ExG=2G−R−B

(16) $$\mathrm{VARI}=\frac{G-R}{G+R-B}$$

(17) $$\mathrm{GLI}=\frac{2G-R-B}{2G+R+B}$$

In these equations, *R*, *G* and *B* represent the pixel values of the red, green and blue channels respectively. The three vegetation-index maps are still spatially registered to the original images so that direct comparison between the model predictions and the spectral responses at the same location is viable. According to Figure 5, smaller vegetation-index values (especially in warm-colored areas) typically imply greater vegetation stress, degradation or mortality. Most of the detection boxes generated by SGM-RTDETR are found in or close to such low-value areas, indicating that the recognized diseased objects are not only spatially well localized but also align with biologically meaningful stress patterns. In contrast to NDVI, which requires multispectral or hyperspectral images, the RGB-based vegetation indices applied in this paper can be calculated using conventional visible band images only. It reduces the cost of data acquisition and renders the method more feasible to perform large-area PWD studies on satellite systems.

### 3.4. Module Effectiveness Experiments

Two modules were considered in the presented work, namely, SVFM and DFRM. The assessment started with SVFM which was introduced in the baseline model as first step and followed by the analysis of DFRM in the same environment. In this way, the effect of each module could be observed through their gradual incorporation into SGM-RTDETR.

#### 3.4.1. Effectiveness of the SVFM Module

To assess the contribution of SVFM, we compared it with several widely used fusion approaches, namely simple concatenation (Concat), Feature-wise Linear Modulation (FiLM), and Cross-Attention fusion [30,31,32]. The detailed quantitative comparison results are reported in Table 2. Experimental results show that the SVFM achieves an mAP@0.5 of 79.71% and an mAP@0.5:0.95 of 35.41% on the test set, outperforming all other compared fusion strategies overall. This indicates that relying solely on simple concatenation or a single interaction mechanism is insufficient to fully exploit the complementary value between visible-band vegetation indices and domain-specific textual semantics, whereas the SVFM, through its hierarchical collaborative design of “input-side VI visual enhancement and decoder input-side textual semantic enhancement”, can more effectively improve the discriminability and robustness of the encoded features [33].

#### 3.4.2. Effectiveness of the DFRM Module

In Table 3, to validate the effectiveness of the DFRM module under complex backgrounds and subtle disease feature scenarios, this paper compares it with typical lightweight attention/feature enhancement methods, including SE, CBAM, and ECA [34,35,36]. The results show that the DFRM achieves an mAP@0.5 of 79.70% and an mAP@0.5:0.95 of 35.61% on the test set, outperforming all compared methods. This demonstrates that the hierarchical strategy of jointly performing channel recalibration, nonlinear feature refinement, and residual enhancement on the encoder memory pathway, compared with single-dimensional attention reweighting, can more effectively activate discriminative channel responses relevant to infected tree detection, thereby improving the model’s perception capability for subtle disease regions under complex forest backgrounds.

### 3.5. Ablation Experiments

In order to analyze the function of each of the proposed modules with respect to detection performance, we incorporated SVFM and DFRM into the RT-DETR baseline one at a time and then assessed the model with both modules applied [37]. The detailed ablation results are summarized in Table 4. The unified evaluation setting achieves a score of 78.01% mAP@0.5 and 33.63% mAP@0.5:0.95 using the baseline model. The addition of the SVFM leads to an increase in those values to 79.71% and 35.41% respectively. The respective metrics when using only DFRM are 79.70% and 35.61%. Combining the two modules further enhances the mAP@0.5 to 80.75%, which is the best result across all the configurations, whereas the value of mAP@0.5:0.95 becomes 35.43%. The findings indicate that SVFM and DFRM are supplementary to each other in enhancing the main detection indicator. Although the dual-module model does not surpass all the single-module settings on mAP@0.5:0.95, it yields the highest mAP@0.5, which better matches the practicality of the primary detection capability in the real-life problem of identifying diseases.

The findings indicate that either SVFM or DFRM can enhance the performance of RT-DETR baseline when implemented independently, but the improvement is not the same. The SVFM-only model achieves 79.71 percent mAP@0.5, and the DFRM-only model achieves 79.70 percent, both greater than the baseline value of 78.01. Combining the two modules causes mAP@0.5 to increase even further, up to 80.75, which is the highest across all configurations. Such a pattern suggests that the two improvement methods can be applied complementarily and they do their job well to enhance the detection of infected trees in complex scene conditions.

The joint model is not the best-performing model on all evaluation metric, and its mAP@0.5:0.95, precision and recall are not always greater than those of the single module variants, though the gaps tend to be minimal in general. It indicates that the greatest benefit of integrating the two modules is enhancing the overall quality of detection instead of maximizing the value of all other secondary measures simultaneously. With the combination of the previous data optimization technique, the optimized training samples offer a better foundation to deal with complex backgrounds, variations in target scales as well as subtle texture differences. In such situations, SVFM can make a greater contribution to semantically enhance, whereas DFRM can make a more effective contribution to locally refine details. The combined effects of both modules are quite complementary: the model exhibits the highest improvement in mAP@0.5 and is relatively stable across the rest of the evaluation metrics, which confirms the utility of the approach to identify PWD-affected trees.

### 3.6. Comparison with State-of-the-Art Methods

#### 3.6.1. SGM-RTDETR Performance on Naro Dataset

SGM-RTDETR was compared with seven representative detectors under the same test conditions, including YOLOv3, YOLOv5s, YOLOv8n, YOLOv9c, YOLOv10n, YOLOv11n, and RT-DETR-L. The quantitative results are summarized in Table 5. Compared with the lightweight YOLO-series models, SGM-RTDETR achieved the highest mAP@0.5 of 0.8074 and a competitive mAP@0.5:0.95 of 0.3543, while maintaining moderate model complexity with 31.987 M parameters, 102.49 GFLOPs, and 30.00 FPS. YOLOv11n had a shorter inference time, but its mAP@0.5 was still lower than that of SGM-RTDETR. SGM-RTDETR has improved the mAP@0.5 of RT-DETR-L without increasing the number of parameters or computational cost significantly. Thus, the new model is more suitable for the trade-off between detection accuracy and runtime speed in PWD recognition on satellite images.

**Figure 6:**
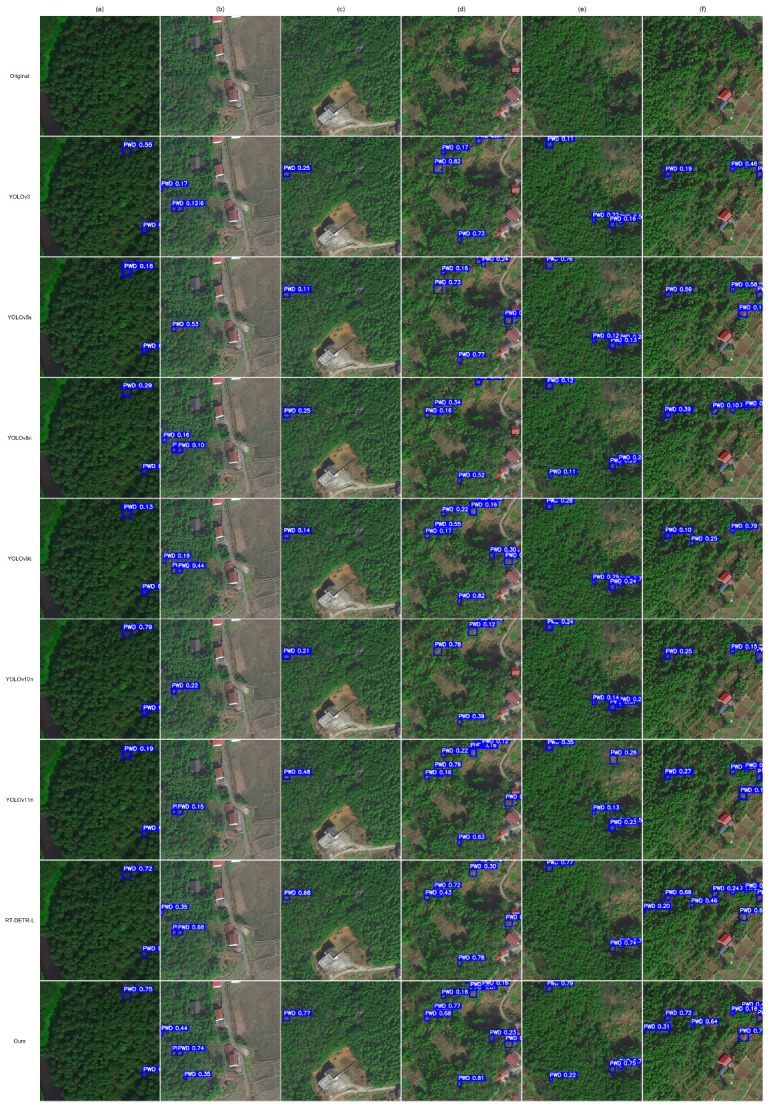
Visual comparison results of different detection models on the Naro dataset. (**a**–**f**) Six representative test samples.

Figure 6 shows the detection results of all models on some representative images in the Naro test set. The six examples chosen were designed to be relatively difficult, with some weak symptoms, small targets, a cluttered forest background, road-forest mixing, and dense candidate regions. The results of some of the baseline models still have missed detections, repeated predictions, unstable confidence scores, or false alarms due to background interference. SGM-RTDETR performs better in the above cases; it has a more stable result, clearer localization, and consistent detection responses. The two are likely related to the function of SVFM and DFRM. SVFM strengthens disease-related feature discrimination by integrating visible-band vegetation cues and CLIP-based text semantics. DFRM then refines the decoder features through channel recalibration and residual enhancement. Overall, both the metric results and the visual comparisons indicate that SGM-RTDETR can better identify PWD-related targets in complex satellite scenes.

#### 3.6.2. Performance of SGM-RTDETR on the Roboflow Dataset

To further assess the performance and generalization of SGM-RTDETR, we used a publicly available PWD detection dataset from the Roboflow platform https://universe.roboflow.com/project-dkq3q/-9pmdt/dataset/8 (accessed on 19 February 2026) [38]. The original dataset contains 4301 training images, including brightness augmentation ranging from −15% to +15%, together with 412 validation images and 202 test images, all at a resolution of 640×640 pixels. To keep the experimental scale manageable while examining how the model generalizes under limited training data, we selected 800 images from the original training set to form a training subset, whereas the validation and test sets were kept unchanged. We screened the images in this dataset and compared SGM-RTDETR against six existing detection methods under identical experimental conditions.

Table 6 shows that SGM-RTDETR delivers the strongest performance on the public PWD dataset among the compared methods. Its mAP@0.5 reaches 83.3%, which is 3.9 percentage points higher than RT-DETR (79.4%) and 0.7 points above YOLOv8n (82.6%), the second-best model. The model also records 80.2% precision, representing a 2.1-point gain over the RT-DETR baseline. Recall reaches 77.6%, which is 0.8 points higher than the baseline. The overall results on the public dataset suggest that the method is good at generalizing and still has a significant improvement over RT-DETR in mAP@0.5.

The performance benefits of SGM-RTDETR on the PWD dataset are due to such factors as: (a) the fact that SGM-RTDETR is based on the RT-DETR architecture and inherits the benefits of the Transformer encoder–decoder structure in the global feature modeling, which allows the better capturing of the global contextual relationship between the PWD-infected areas and the healthy vegetation around them; (b) the Semantic–Visual Fusion Module (SVFM) that is based on the Stackelberg game, through the integration of CLIP textual semantics information, increases the semantic comprehension of the model to the PWD features, allowing the more precise distinction between dead trees of pine and other visually alike land cover features (bare soil, dead branches, etc.); (c) the vegetation indices (ExG, VARI, GLI), which are based on RGB, act as additional input sources, offering the model a direct quantitative insight into the vegetation health state, making up the lack of vegetation stress recognition ability of pure RGB images; and (d) the optimization strategy of DFRM, i.e., random subgroup selection and adaptive feature refinement, improves the learning ability of the model in relation to hard samples during training, thus increasing the strength of the detection. All these findings indicate that SGM-RTDETR does not only perform well in the self-constructed dataset but also on the public dataset, which suggests that the given approach can be considered effective and generalizable.

#### 3.6.3. Performance of SGM-RTDETR on the PDT Dataset

To further evaluate the cross-dataset generalization ability of SGM-RTDETR, we introduced the publicly released PDT dataset from the PDT_CWC_YOLO-DP project https://github.com/RuiXing123/PDT_CWC_YOLO-DP (accessed on 27 May 2026). According to the dataset authors, the PDT dataset is the first high-precision UAV dataset specifically designed for the targeted detection of tree pests and diseases, and it was collected in real operational environments to fill the gap of dedicated datasets in this field. Compared with satellite remote sensing imagery, UAV-acquired imagery has different imaging perspectives, target scales, and background distributions [39]. Therefore, evaluation on the PDT dataset provides an additional cross-source and cross-platform test for the proposed method. The original PDT dataset contains 4301 training images, 412 validation images, and 202 test images. Under identical training and evaluation settings, SGM-RTDETR was compared with seven existing detectors.

Table 7 shows that SGM-RTDETR achieves the best overall performance on the PDT dataset among the compared methods. Its mAP@0.5 reaches 79.81%, which is 2.55 percentage points higher than RT-DETR (77.26%) and 0.94 points higher than YOLOv11n (78.87%), the second-best model in this metric. SGM-RTDETR achieves the highest precision of 80.79% and recall of 79.64%, and thus can detect tree disease targets in UAV images with a relatively high degree of both accuracy and sensitivity. Its mAP@0.5:0.95 is not the highest among all models, but it is still 2.61 percentage points better than RT-DETR. These results indicate that SGM-RTDETR can maintain competitive performance on an independent public UAV dataset and shows robustness to variations in image sources and acquisition conditions.

The PDT results show that the following parts help improve SGM-RTDETR: global context modelling, semantic-guided feature enhancement, vegetation-index cues and adaptive decoder refinement. SGM-RTDETR is not only for using RGB images but also incorporates both semantic information and vegetation-related responses in its disease identification of trees in complex UAV images. The PDT experiment further shows that the model can transfer to data beyond the self-constructed Naro dataset and the existing Roboflow PWD dataset.

## 4. Discussion

The three problems in the existing detectors of satellite-based detection of pine wilt disease (PWD) were addressed in this study to improve them. First, the disease-related semantic knowledge is not utilized. Second, visible-band vegetation cues are often only weakly combined. Third, the specific traits of the disease are still hard to express. Based on the previous studies of forest disease monitoring and remote sensing object detection, spectral changes, background contexts, and object-level spatial characteristics can all serve as indicators of early-stage or minor symptoms of vegetation stress. Many general-purpose detectors have only learned from RGB images thus far. Therefore, if the symptoms of PWD are mild, the diseased areas are small, or the surrounding forest environment is complex, it may not be suitable for this method. To address the above problems, we design SGM-RTDETR within the RT-DETR framework by integrating vegetation-index-enhanced visual cues, CLIP-based semantic guidance and feature refinement.

**Figure 7:**
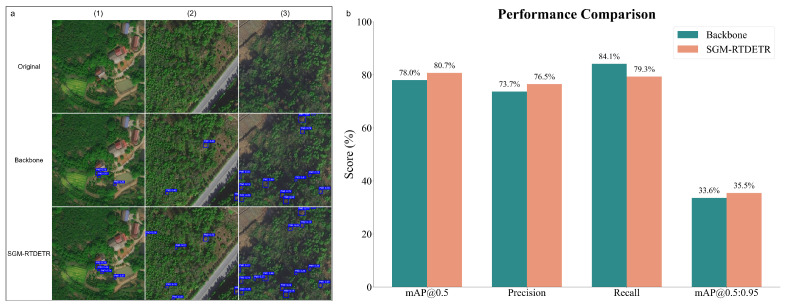
Performance comparison between the baseline model and SGM-RTDETR. (**a**) Visualization results; (**b**) Statistical data.

As shown in Figure 7b, SGM-RTDETR achieved an mAP@0.5 of 80.75% under the same experimental conditions, and it was higher than the 78.01% obtained by RT-DETR-L. Therefore, it is proposed that a two-module design will be used to recognise diseased pine crowns. In accordance with the above research results, auxiliary information at the domain level can enhance the precision of remote sensing detection models. SVFM adds improvements to vegetation indices and text-based semantic information in our framework as additional cues for distinguishing PWD-affected crowns from complex backgrounds. DFRM strengthens the representation of local texture and structural variations in the memory feature extraction stage of the decoder. The two modules are not the same but work together. SVFM expands semantic–visual discrimination, and DFRM increases feature consistency for local stabilization.

Figure 7a shows some more typical examples. SGM-RTDETR performs better in areas with mild disease symptoms, small infected crowns, a mixed forest-road-building background, and a high density of candidate objects. The above visual observations are consistent with the numerical results, and it can be concluded that the new modules improve the accuracy of detection in complicated satellite images. Detection based only on RGB appearance is not sufficient; therefore, visible-band vegetation indices that contain information about canopy color variation and thus are related to PWD symptoms, such as VARI, GLI, and ExG, should also be added. At the same time, text-guided semantic information helps the model pay more attention to disease-related visual patterns and is no longer solely based on low-level appearance features. Based on previous research, the above results suggest that domain knowledge and semantic guidance can be used to improve forest disease detection in remote sensing images.

The experiments also have some deficiencies. As shown in Table 5, SGM-RTDETR reached 35.43% for mAP@0.5:0.95, but it was not the highest among all the other methods. Therefore, it can be seen that localization accuracy at a high IoU threshold has not improved yet. SVFM and DFRM are likely to improve semantic separation and general detection stability, but they do not specifically address the problem of bounding box regression. In other words, the current model gives more weight to target recognition and has not yet strengthened precise box localization sufficiently. Another problem is that, at present, the way it works is based only on RGB images and the visible-band vegetation indices derived from them. In actual forest conditions, the cause of canopy change may be PWD; however, other reasons such as drought, shade, variations in tree species, seasonal changes and other disturbances can also lead to such a change. Therefore, under certain field conditions, the model may still misclassify infected trees as healthy or stressed. This limitation should therefore be considered when the model is applied to operational forest monitoring.

Future work will focus on several improvements. First, a bounding-box refinement module can be added to improve the accuracy of localization, such as cascade regression or deformable convolution. Second, the loss function can be modified to increase the penalty weight for high-IoU regions and thus improve the accuracy of box prediction. Thirdly, the semantic information generated by SVFM could be more closely coupled with the bounding-box regression branch to provide semantic priors for both classification and localization. Future studies should also incorporate near-infrared or multispectral imagery rather than relying only on RGB data, and these extra spectral bands will be used to find more reliable cues for distinguishing PWD-related stress from non-disease stress compared to just RGB data. Multi-scale feature fusion also needs to be improved, and the location of disease in different sizes should also be enhanced.

Overall, SGM-RTDETR shows that semantic–visual fusion and decoder feature refinement can improve PWD target recognition in satellite imagery without greatly increasing model complexity. The framework still needs further improvement in high-IoU localization and generalization across multiple data sources. Even so, it shows practical potential for large-scale forest disease screening. Therefore, SGM-RTDETR is more suitable as an auxiliary tool for remote sensing-based early warning and monitoring of PWD, rather than as an independent or final basis for field diagnosis.

## 5. Conclusions

This study focused on three problems in satellite-based PWD detection: the limited use of disease-related semantic knowledge, the insufficient fusion of visible-band vegetation indices, and the weak description of fine disease details. To deal with these problems, we developed SGM-RTDETR, a dual-module detector built on RT-DETR. In this model, SVFM combines vegetation-index-driven visual enhancement with CLIP-guided textual semantics through a Stackelberg-inspired fusion strategy. DFRM is placed before the decoder to refine encoder memory features and improve the representation of subtle disease-related patterns.

SGM-RTDETR achieved an mAP@0.5 of 80.75% on the Naro test set and surpassed the RT-DETR-L baseline. The above indicates the value of a two-module collaborative design. Vegetation-related cues, semantic guidance and feature refinement can be used together to improve the recognition accuracy of PWD-affected tree crowns in complex satellite images. The main contribution of this paper is, therefore, a remote sensing detection method that improves the target recognition for diseases while maintaining a reasonable balance between accuracy and model complexity.

There are still some deficiencies, including the need to improve the accuracy of bounding-box localization in the model under a stricter IoU requirement. It may also confuse PWD symptoms with other causes of canopy discoloration in some forest conditions. Future work will thus consider bounding-box refinement, cross-region validation, time-series monitoring, and multi-source data fusion. Near-infrared and multispectral imagery could be incorporated to provide richer spectral information. The above extensions will enhance the robustness and real-world application value of SGM-RTDETR for large-scale forest disease monitoring and early warning.

## Figures and Tables

**Table 1:** Hardware and software environment settings.

Category	Component	Specification
Hardware	CPU	Intel Core i9-12700K
	RAM	32GB DDR5
	GPU	NVIDIA GeForce RTX 3060 (6 GB)
Software	OS	Windows 11
	Python	3.9.7
	CUDA Toolkit	12.1
	PyTorch	2.4.1
	Ultralytics	8.3.241

CPU: Central Processing Unit, RAM: Random Access Memory, GPU: Graphics Processing Unit, OS: Operating System.

**Table 2:** Experimental findings on the effectiveness of SVFM.

Methods	mAP@0.5 (%)	Precision (%)	Recall (%)	mAP@0.5:0.95 (%)
Concat	78.7	74.6	75.2	35.5
FiLM	78.3	75.8	76.0	33.7
Cross-Attention	78.9	74.2	81.3	34.3
SVFM	79.71	78.58	76.92	35.41

**Table 3:** Experimental findings on the effectiveness of DFRM.

Methods	mAP@0.5 (%)	Precision (%)	Recall (%)	mAP@0.5:0.95 (%)
SE	78.76	72.21	75.96	35.11
CBAM	78.25	74.49	78.85	34.85
ECA	78.82	73.64	79.22	35.23
DFRM	79.70	76.20	80.77	35.61

**Table 4:** Ablation, ✓ means used/included, × means not used/not included experiment.

SVFM	DFRM	mAP@0.5 (%)	mAP@0.5:0.95 (%)	Precision (%)	Recall (%)
×	×	78.01	33.63	73.71	84.13
✓	×	79.71	35.41	78.58	76.92
×	✓	79.70	35.61	76.20	80.77
✓	✓	80.75	35.43	76.51	79.33

**Table 5:** Quantitative comparison of SGM-RTDETR with representative detectors on the Naro dataset.

Model	R	mAP@0.5 (%)	mAP@0.5:0.95 (%)	Params (M)	GFLOPs	FPS
YOLOv3	0.716	0.767	0.403	103.7	283.3	24.17
YOLOv5s	0.668	0.729	0.386	9.2	24.2	40.24
YOLOv8n	0.639	0.733	0.371	3.2	8.9	45.11
YOLOv9c	0.712	0.737	0.373	25.6	104.0	24.18
YOLOv10n	0.702	0.752	0.372	2.8	8.7	40.16
YOLOv11n	0.731	0.790	0.397	2.6	6.6	56.03
RT-DETR-L	0.8413	0.7801	0.3363	31.986	103.43	29.63
Ours	0.7933	0.8074	0.3543	31.987	102.49	30.00

**Table 6:** Comparison with other models on the PWD dataset.

Model	mAP@0.5 (%)	Precision (%)	Recall (%)	mAP@0.5:0.95 (%)	Params (M)	FLOPs (G)
YOLOv3	81.9	79.3	73.9	38.3	103.7	282.2
YOLOv5s	82.5	75.6	75.9	39.2	9.1	23.8
YOLOv8n	82.6	79.0	72.9	39.4	3.0	8.1
YOLOv9c	82.4	79.0	75.4	38.5	25.3	102.3
YOLOv10n	81.2	78.1	73.5	38.7	2.3	6.5
YOLOv11n	81.6	77.1	74.5	39.2	2.6	6.3
RT-DETR	79.4	78.1	76.8	35.2	32.0	103.4
SGM-RTDETR	83.3	80.2	77.6	37.3	32.0	103.4

**Table 7:** Quantitative comparison of SGM-RTDETR with representative detectors on the PDT dataset.

Model	mAP@0.5 (%)	Precision (%)	R (%)	mAP@0.5:0.95 (%)	Params (M)	GFLOPs
YOLOv3	78.15	80.19	75.38	38.45	103.67	282.21
YOLOv5s	75.80	79.90	79.48	39.10	9.11	23.83
YOLOv8n	76.30	81.62	74.59	38.16	3.01	8.09
YOLOv9c	76.93	81.29	74.47	40.89	25.32	102.32
YOLOv10n	77.80	78.04	72.95	39.43	2.27	6.53
YOLOv11n	78.87	75.79	78.42	39.35	2.58	6.31
RT-DETR	77.26	79.54	72.07	33.19	31.99	103.43
SGM-RTDETR	79.81	80.79	79.64	35.80	32.0	108.60

## Data Availability

The data related to the findings of this research are available from the corresponding author upon reasonable request. The data are not publicly available due to privacy, ethical, and data-use restrictions associated with field survey records and geospatial information on pine wilt disease occurrence.
